# Sarcomatous transformation of IDH-mutant astrocytoma matching to methylation class oligosarcoma following embolization, a case report

**DOI:** 10.1186/s40478-024-01908-7

**Published:** 2024-12-20

**Authors:** Ryan Landvater, Arushi Tripathy, Edwin Nieblas-Bedolla, Lina Shao, Kyle Conway, Wajd Al-Holou, Sean P. Ferris

**Affiliations:** 1 Department of Pathology, University of Michigan Medical School, 2800 Plymouth Road, Ann Arbor, MI 48109 USA; 2 Department of Neurosurgery, University of Michigan Medical School, 1500 E Medical Center Drive, Ann Arbor, MI 48109 USA

## Abstract

The mesenchymal transformations of infiltrating gliomas are uncommon events. This is particularly true of IDH-mutant astrocytomas and oligodendrogliomas, in which mesenchymal transformation is exceedingly rare. oligosarcoma is a newly recognized methylation class (MC) that represents transformed 1p/19q co-deleted oligodendrogliomas, but recent studies indicate it may be non-specific. Herein we report the diffuse sarcomatous transformation of a multifocal recurrent astrocytoma from a precursor IDH-mutant astrocytoma, CNS WHO grade 3, in a young patient following embolization therapy and matching to MC oligosarcoma. The sarcomatous recurrence and original tumor showed identical 17q breakpoints with loss of heterozygosity of *TP53*. Both lack the defining 1p/19q co-deletion or copy-neutral heterozygosity of an oligodendroglioma and oligosarcoma. The findings in this case report both contribute to the apparent heterogeneity of the novel MC oligosarcoma and describe a second reported mesenchymal transformation of an IDH-mutant astrocytoma.

**Supplementary Information:**

The online version contains supplementary material available at 10.1186/s40478-024-01908-7.

## Introduction

Gliosarcoma is a rare variant of GBM, representing approximately only 2% of all *IDH*-wildtype GBMs and characterized by biphasic histological features including glial and sarcomatous elements. Gliosarcoma is exclusively *IDH*-wildtype [1]. These tumors may arise de novo or develop from the sarcomatous transformation of a precursor *IDH*-wildtype glioblastoma; however, rare sarcomatous transformations of *IDH*-mutant gliomas have been reported [2]. The transcriptional and epigenetic changes underlying sarcomatous transformation are incompletely understood [3, 4]. Although gliosarcomas are generally treated in the same manner as GBMs (with maximal surgical resection, followed by adjuvant temozolomide and radiotherapy), the rarity of these mesenchymal gliomas poses prognostic and therapeutic challenges [5, 6]. Few cases highlighting the change in pathologic features over time have been reported [7].

While GBM—and gliosarcoma by extension—is thought to be astrocytic in lineage, there are reports of tumors where the glial component of the sarcoma is ependymal or oligodendroglial [8]. Oligodendrogliomas are infiltrating gliomas that, by definition, possess mutations in the *IDH1* or *IDH2* genes and whole arm co-deletion of chromosomes 1p and 19q. They also typically possess *TERT* promoter mutations, lack mutations in the *ATRX* gene expression, and lack *TP53* mutations [9]. The additional presence of sarcomatous features, which include a variety of patterns such as areas of bipolar fusiform cells with intersecting bundles and fascicular growth, are proposed to define an oligosarcoma diagnosis and confer a distinctly more aggressive clinical course when compared to oligodendrogliomas that lack these morphologies [10]. In the event of diagnostic ambiguity, genome-wide DNA methylation profiling can aid in establishing the diagnosis with a greater degree of certainty, as oligosarcomas have been shown to be epigenetically distinct [10].

Genome-wide DNA methylation profiling with use of a machine learning classifier (methylation profiling) is a recently developed molecular technique that can be useful for brain tumor diagnosis [11] and recently new MC named oligosarcoma was described [10]. Suwala et al. reported that 50% of the tumors matching to the novel MC oligosarcoma developed from a known oligodendroglial precursor and 50% are hypothesized to have developed de novo. While most MC oligosarcoma tumors demonstrated chromosome 1p/19q-codeletion, 5 cases (20%) had no evidence of 1p/19q co-deletion, but showed CnLOH of chromosomes 1p and 19q [10]. The authors proposed a diagnosis of oligosarcoma requires “(a) sarcomatous histology, (b) *IDH*-mutation and (c) *TERT* promoter mutation and/or 1p/19q codeletion, or, in unresolved cases, on its characteristic DNA methylation profile” [10]. Since this initial report, there have been at least two reports suggesting that MC oligosarcoma may encompass non-oligodendroglial entities, including a GBM and an *IDH*-mutant astrocytoma [7, 12].

Herein we report a second case of recurrent *IDH*-mutant astrocytoma showing sarcomatous transformation and matching to MC oligosarcoma following radiation and embolization therapy. Our report expands our understanding of these tumors by describing comprehensive molecular data showing the clonal progression of the tumor. Methylation profiling performed on the sarcomatous component of the recurrence matched to MC oligosarcoma with high confidence, while the methylation profiling performed on the initial resection specimen and on the micro-dissected glial component of the recurrent tumor both matched to MC astrocytoma, low-grade, with high confidence. There was neither evidence of whole arm 1p/19q-codeletion nor post-deletion homologous recombination with CnLOH by CMA in either the initial resection specimen or in the sarcomatous recurrence. This case report aims to contribute to the understanding of sarcomatous recurrence in low-grade astrocytic gliomas, to contribute to the understanding of MC oligosarcoma, and to highlight the clinical implication in one young patient as it pertains to clinical manifestations, diagnostic approach, epigenetic transformation over time, and ultimately treatment.

## Case presentation

### Clinical

**Fig. 1:**
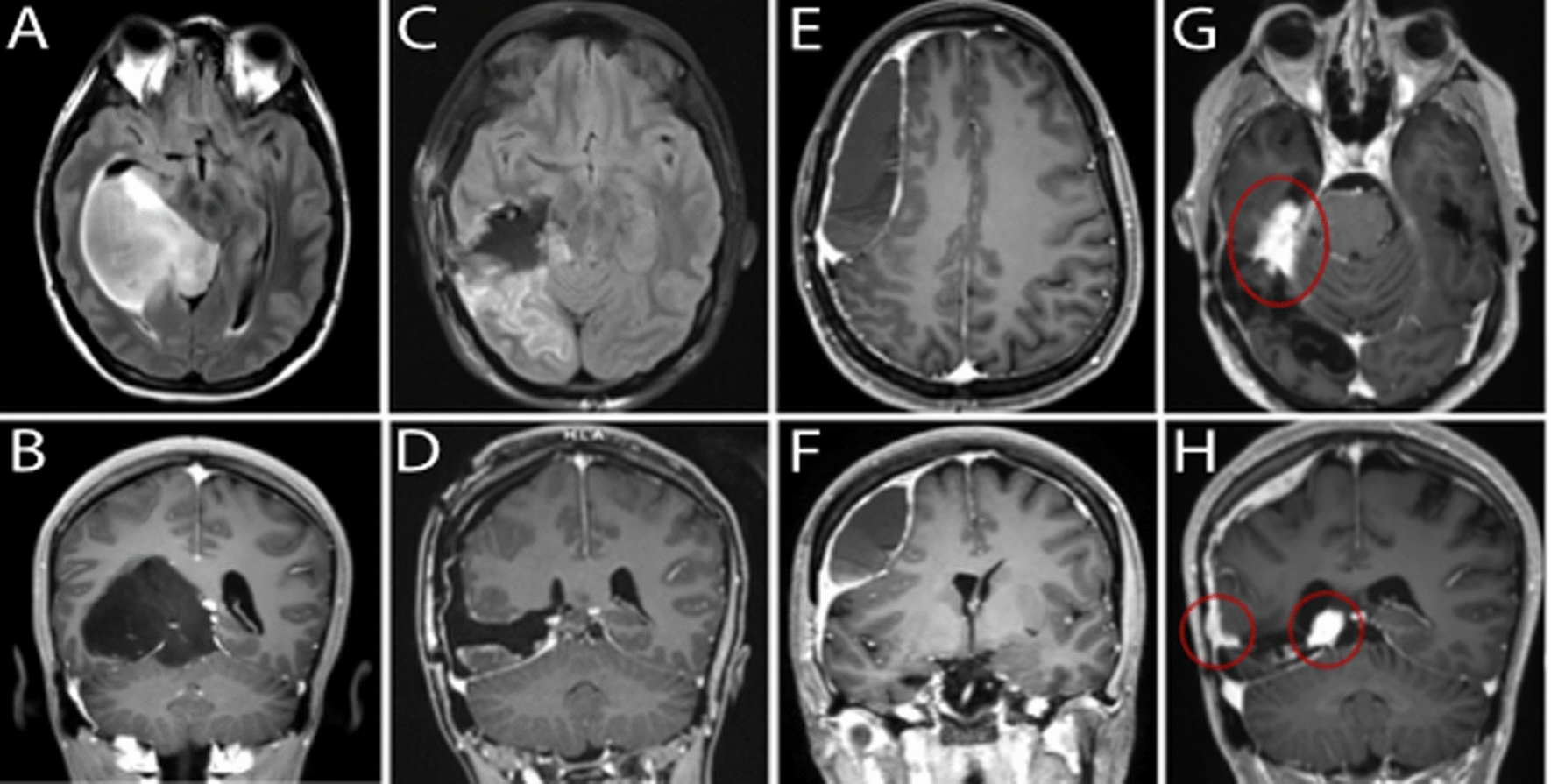
Magnetic resonance imaging over the course of treatment. Axial FLAIR (**A**) and coronal T1 post-contrast (**B**) images of tumor at time of presentation. Axial FLAIR (**C**) and T1 post-contrast (**D**) images following operative resection. Axial (**E**) and T1 post-contrast (**F**) images demonstrating subsequent formation of peripherally enhancing, hypodense extra-axial fluid collection with underlying mass effect and right to left midline shift. Axial (**G**) and coronal (**H**) T1 post-contrast images demonstrating multifocal recurrence including along the tentorium and convexity

A 26-year-old female with history of migraine headaches presented with a 6-month history of intermittent 30 min episodes of left hemiparesthesia, typically preceded by kaleidoscopic vision changes. An MR image demonstrated a large infiltrative T2 hyperintense intrinsic tumor involving the right temporal and occipital lobes, herniating over the medial tentorial edge and causing mass effect on the brainstem (Fig. 1A, B). The tumor showed no evidence of abnormal enhancement or hyperperfusion. Given the size and complexity of a tumor involving the supratentorial and infratentorial fossae and multiple lobes, a staged resection was offered. An incision was fashioned to allow for both planned stages. A stage one right temporo-occipital craniotomy allowed resection of the posterior tumor using the lateral ventricle as a superior landmark, and a subtemporal transtentorial approach to resect tumor compressing the brainstem. Expectedly, the patient sustained post-operative incomplete left-sided homonymous hemianopsia. One month later, a stage two craniotomy resected the remaining anterior component resulting in gross total radiographic resection (Fig. 1C, D).

Surgical resection was followed by standard protocol radiation and temozolomide, however three years later, the patient presented with worsening headaches and new subtle left lower extremity weakness. MR imaging now demonstrated atypical dural enhancement and what seemed to be a benign underlying right convexity subdural hematoma (Fig. 1E, F). Given her mild symptoms, evacuation was deferred in favor of right middle meningeal artery embolization resulting in temporary symptomatic relief and radiographic improvement in the hematoma size. However, 8 months following embolization, the patient was found to have focal areas of progressive diffuse dural thickening and enhancement along the convexity dura and tentorium (Fig. 1G, H), concerning for tumor recurrence. She underwent repeat craniotomy for resection and biopsy followed by re-irradiation of all enhancing areas over the dura and tentorium and chemotherapy (cyclic lomustine).

The patient discontinued Lomustine six months after the last resection and started Vorasidenib nine months following the last resection. At that time (9 months), the patient showed MR findings suspicious for progression. At ten months, there was clear progression on MR exam with peripheral nodular enhancement of the resection cavity and along the right subdural space with subdural thickening and extension.

### Pathology

#### Histopathology and next generation sequencing

**Fig. 2:**
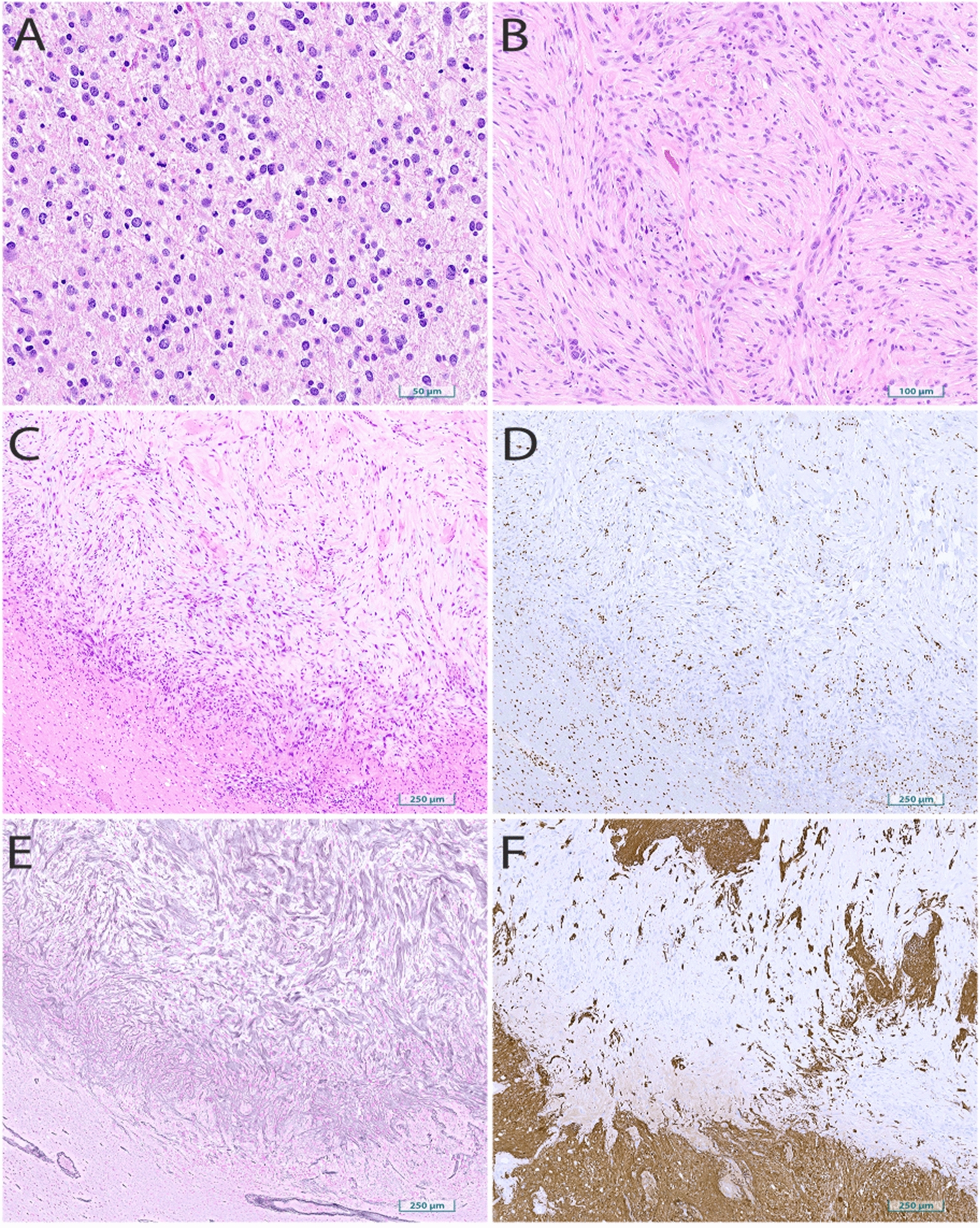
Histology of initial resection and tumor recurrence. Hematoxylin and eosin-stained sections of **A** the initial resection demonstrating an infiltrative astrocytic neoplasm with irregular nuclear morphology and mitotic activity and **B**–**C** the re-resection specimen showing sarcomatous morphology. Stains on the re-resection specimen demonstrates **D** ATRX loss in the tumor and with retained expression in surrounding parenchyma, **E** reticulin fiber staining within the tumor, and **F** GFAP loss in the tumor with retained surrounding expression

Initial diagnosis demonstrated a mitotically active infiltrating glioma with nuclear pleomorphism but without sarcomatous morphology (Fig. 2A). Up to 5 mitoses were identified in 10 high-powered fields. GFAP IHC was diffusely positive. ATRX IHC was lost in tumor cells. IHC for p53 did not show aberrant over-expression. *IDH1*-R132H mutant-specific IHC was negative. Solid tumor NGS panel (Illumina NexSeq 550) on genomic DNA extracted from FFPE tissue showed an IDH1 exon 4 c.394C > T (R132C) point mutation. TEMPUS NGS confirmed the IDH1 mutation and showed ATRX and ARID1B frame-shift loss of function mutations. Characteristic oligodendroglioma mutations were not present (*TERT, FUBP1, CIC*). A *MSH6* missense mutation c.2407G > C (p.D803H) of uncertain significance (VUS) was also identified via TEMPUS and the tumor was found to be microsatellite stable by IHC proxy with retained expression of MSH6. Germline testing for Lynch syndrome was not performed. The final diagnosis was anaplastic astrocytoma, *IDH*-mutant, CNS WHO grade 3 based on diagnostic criteria at the time.

Histology from the recurrent dural-based resection was overwhelmingly sarcomatous (>95% of overall tumor) with tumor morphologically characterized by spindled cells containing atypical nuclei embedded in a myxoid and collagenous background (Fig. 2B, C). An adjacent focus of glial tumor component was also present. GFAP and OLIG2 were not expressed in the sarcomatous regions of the tumor but positive within the glial component. *ATRX* was lost in both the sarcomatous and glial components. p53 was not overexpressed. Sarcomatous regions of the tumor were reticulin fiber rich. There was extensive strong positivity for muscle-specific actin in the sarcomatous regions. A combined GFAP/Ki67 stain showed rare, atypical GFAP-positive proliferating tumor cells in the glial component. The full IHC profile is included in Table 1. NGS was performed on the sarcomatous component, which contained the same IDH1-R132C point mutation as the initial resection specimen.

**Table 1:** Full immunohistochemical profile

Marker	Sarcomatous morphology	Gliomatous morphology
***Recurrent tumor***		
GFAP	Negative	Positive
IDH1-R132H	Negative	Negative
Reticulin	Reticulin-fiber rich	Negative
Neurofilament	Excluded	Infiltrative growth
ATRX	Loss of expression	Loss of expression
OLIG2	Negative	Positive
p53	$\hspace{0.17em}<\hspace{0.17em}$10% of tumor nuclei	$\hspace{0.17em}<\hspace{0.17em}$10% of tumor nuclei
MS Actin	Extensive strong positivity	Negative
Ki-67	$\hspace{0.17em}\approx \hspace{0.17em}$40%	
GFAP/Ki-67		Atypical dual positive cells
MMR proteins	MSH2, MSH6, MLH1, PMS2 retained	MSH2, MSH6, MLH1, PMS2 retained
***Initial resection***		
GFAP		Diffusely positive
IDH1-R132H		Negative
H3-K27me3		Retained expression
ATRX		Loss of expression
p53		Negative
Ki-67		$\hspace{0.17em}\approx \hspace{0.17em}$10%
MMR proteins		MSH2, MSH6, MLH1, PMS2 retained

#### Chromosomal microarray and genome-wide DNA methylation array studies

Genomic DNA was extracted, and chromosomal microarray studies were performed to evaluate the clonal progression of the tumor molecular features on the following two specimens: (1) the original tumor and (2) the sarcomatous component of the glial tumor. Genome-wide DNA Methylation was performed on the following three specimens: (1) the original tumor and (2) the sarcomatous component of the glial tumor; and (3) the glial component of the recurrent tumor.

CMA studies were performed on the OncoScan CNV Plus microarray assay from ThermoFisher Scientific and the results were interpreted using the ChAS software. Copy-number alterations greater than 50–100 kb in cancer genes or greater than 3 MB outside clinical oncology significant regions, and CnLOH greater than 10 MB were reported. Genome-wide DNA Methylation Array Studies were performed on the Infinium EPIC-8 v2.0 HD Methylation Beadchip. Interpretation of the genomic DNA methylation results were completed on v12.8 of the DKFZ/Heidelberg classifier [11].

**Fig. 3:**
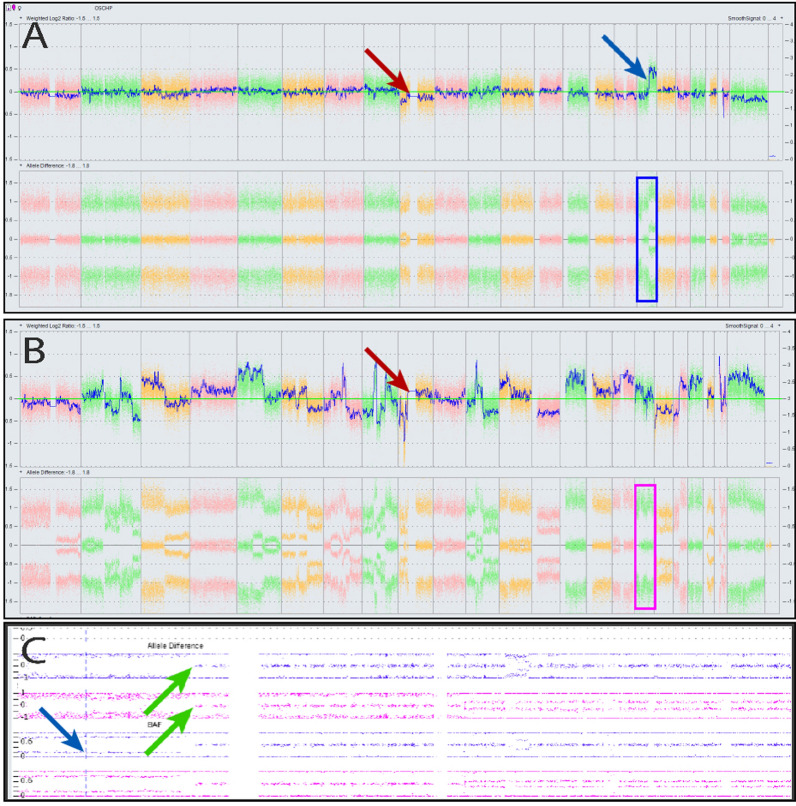
Chromosomal Microarray of initial and recurrent tumor. Copy number plots from chromosome 1p (left) to Y (right) for the initial resection (**A**) and recurrence (**B**). The initial resection (**A**) shows loss of chromosome 9/CDKN2A/B (red arrow) and LOH at chromosome 17q, including the TP53 locus (blue arrow). No 1p/19q co-deletion was observed. Recurrent tumor (**B**) showed a complex karyotype with retained 17q LOH breakpoint but no co-deletion nor LOH at 1p/19q. Expanded chromosome 17 plots (**C**), which correspond with blue and pink boxes in **A** and **B**, showed loss of heterozygosity with the same breakpoint (green arrow) in both initial sample (blue) and recurrent sarcomatous tumor (pink)

CMA results from the initial resection showed loss of chromosome 9, including the *CDKN2A/B* locus, chromosome 17p CnLOH (Fig. 3A), 17q gain, and loss of chromosome X. There was neither co-deletion of chromosomes 1p/19q nor was there CnLOH in those chromosome arms (Fig. 3A). The sarcomatous component of the recurrence demonstrated a complex karyotype with abnormalities involving every chromosome, but notable for the same 17p CnLOH (Fig. 3B, C), which supports the clonal relationship between the sampled initial resection and sampled recurrence. In addition, the sarcomatous component did not show 1p/19q co-deletion, although there was LOH of 1p and gain of 19q. Loss of heterozygosity was seen at the IDH1 gene locus; however, there is no definite evidence to support gross copy number alterations at this locus (Supplement 1).

DNA Methylation results showed the initial resection matched with high confidence to methylation class astrocytoma, *IDH*-mutant, low-grade (0.89) with the minor subset class oligodendroglioma, *IDH*-mutant, 1p/19q co-deleted (0.11). Methylation profiling of the glial component of the recurrence matched with high confidence to methylation class astrocytoma, *IDH*-mutant, low-grade (0.99); however, the sarcoma matched with high confidence to the MC oligosarcoma, *IDH*-mutant, with a final confidence score of 0.90. The full methylation classifications for the separate resection specimens and different histologic morphologies are detailed in Table 2.

**Table 2:** Methylation classes as determined by the DKFZ/Heidelberg classifier v12.8

Level	Predicted classification	Classifier score
*Initial resection*		
Super Family	Adult-type diffuse gliomas	0.99
Family	Diffuse glioma, *IDH*-mutant	0.99
Subclass	Astrocytoma, *IDH*-mutant,	0.89
	Lower grade	
	Oligodendroglial type	
Subclass	Oligodendroglioma, *IDH*-mutant,	0.11
	1p/19q co-deleted	
*Recurrent resection*		
*Gliomatous morphology*		
Super family	Adult-type diffuse gliomas	0.99
Family	Diffuse glioma, *IDH*-mutant	0.99
Class	Diffuse glioma, *IDH*-mutant,	0.99
	Astroglial type	
Subclass	Astrocytoma, *IDH*-mutant,	0.99
	Lower grade	
*Recurrent resection*		
*Sarcomatous morphology*		
Super family	Adult-type diffuse gliomas	0.95
Family	Diffuse glioma, *IDH*-mutant	0.91
Class	Diffuse glioma, *IDH*-mutant,	0.91
	Oligodendroglial type	
Subclass	Oligosarcoma, *IDH*-mutant	0.90

The initial resection matched to astrocytoma, IDH-mutant, with an unusual result from the classifier returning a minor minor degree of oligodendroglioma (top). The recurrent tumor matched with high confidence to astrocytoma, MC IDH-mutant, low-grade, in the glial component (middle) and MC Oligosarcoma, IDH-mutant, in the sarcomatous component (bottom)

## Discussion

Although sarcomatous transformations of *IDH*-wildtype glioblastoma into gliosarcomas, and oligodendrogliomas into oligosarcomas, have been well described for decades [9, 13, 14], the diagnosis of *IDH*-mutant astrocytoma with sarcomatous differentiation has only rarely been published [7, 15]. The epigenetic profiles of glioblastoma and oligosarcoma are distinct entities with unique epigenetic clusters; however evidence has shown these MCs may not be entirely specific. Such classifiers may aberrantly mis-classify cases, as demonstrated by a recent case report in which an *IDH*-wildtype gliosarcoma was classified as an oligosarcoma [12]. This raises questions about the specificity of MC oligosarcoma and the genetic underpinnings that define these sarcomatous glial tumors.

Our index patient’s *IDH*-mutant astrocytoma was treated with standard of care gross-total resection and adjuvant radiation with temozolomide. When the patient presented 3 years later, the initial impression was of development of a likely benign subdural hematoma. This was initially managed conservatively with embolization, which improved symptoms and the size of the lesion; however, the dramatic and diffuse dural and tentorial-based sarcomatous recurrence several months later raises the suspicion that this represented early tumor recurrence and further may represent treatment-related tumor evolution [16]. As part of the right convexity tumor bed hematoma intervention, recurrent tumor may have been embolized, an unusual clinical circumstance as embolization is not standard treatment for glioma. Given that in-vivo mouse-model gene expression profiles strongly associated a subset of mesenchymal tumors with hypoxia-response gene expression, which suggests a linkage between mesenchymal tumors and hypoxia (so called ‘mesenchymal-like hypoxia-dependent’ or ‘MES2’ tumors) [17, 18], we questioned whether embolization may have had any causative role in the higher-grade mesenchymal transformation of this *IDH*-mutant astrocytoma. Without any other reported examples of such an occurrence, this question remains speculative but may represent a reasonable subject of future research.

The sarcomatous portion of the recurrence is distinctly astrocytic and clonally derived from the initial *IDH*-mutant astrocytoma, as demonstrated by an identical 17p CnLOH break-point and IDH1-R132C point mutations. Loss of heterozygosity at the IDH1 locus was noted without any definite copy-number alterations of the IDH1 locus. Despite the recurrence sarcomatous component’s complex copy-number plot, there was no evidence of co-deletion or CnLOH involving the 1p/19q arms. In the context of retained 1p and 19q arms, this lack of CnLOH is highly significant in this case because the literature shows oligosarcoma may demonstrate CnLOH of 1p/19q due to reduplication of the these chromosomal arms. Consequently, the sarcomatous recurrences demonstrate CnLOH on CMA as a result of homologous recombination of 1p and 19q [10]. These features define the clonal relationship between the sarcomatous tumor and precursor glioma and strongly support the absence of 1p/19q co-deletion at any point in the tumor progression.

The main limitations of this work pertain to the generalizability of our findings—few similar cases have been described in the literature and we have no reference tumors that matched to MC Oligosarcoma with oligodendroglial features, as described in Suwala et al., within our institution to function as a molecular comparison for these results. With such limited case numbers, there is an intrinsic risk that limited patterns and conclusions may become overly generalized. We have avoided making strong conclusions and made every effort to characterize the molecular features of this case with the exclusion of alternative explanations for our findings.

## Conclusion

We describe a recurrent astrocytoma, *IDH*-mutant, matching to MC oligosarcoma in its sarcomatous component. This case is the second such reported following the work of Liu et al., 2024 [7] and expands upon their work by describing comprehensive molecular characterization describing the clonal progression in the glial and sarcomatous components. We recognize the true incidence of such cases may be under-represented in the literature as access to tools that aid in this diagnosis, specifically genome-wide methylation, are still limited. This case describes vital clinical and pathologic features, and demonstrate that *IDH*-mutant astrocytomas with a sarcomatous component may match to MC oligosarcoma despite diverging from the proposed diagnostic criteria by Suwala et al*.* of Oligosarcoma [10]. While the majority of reported MC oligosarcoma tumors arise from oligodendroglial precursors, these exceedingly uncommon *IDH*-mutant astrocytomas with sarcomatous components may represent a clinical and diagnostic unknown for the practicing neuropathologist and neurosurgeon, alike.

## Supplementary Information

Additional file 1.

## Data Availability

All de-identified genomic data, OncoScan CNV Plus microarray results and genome-wide DNA methylation array results (IDAT files) will be made available from the corresponding author on reasonable request.

## Abbreviations

ATRX: Alpha-thalassemia/mental retardation, X-linked
CMA: Chromosomal microarray
CnLOH: Copy-neutral loss of heterozygosity
FFPE: Formalin-fixed paraffin-embedded
GBM: Glioblastoma
IDH: Isocitrate dehydrogenase
IHC: Immunohistochemical staining
LOH: Loss of heterozygosity
MC: Methylation class
NGS: Next generation sequencing
TERT: Telomerase reverse transcriptase
