# Supplementary figures and images for: Sarcomatous transformation of IDH-mutant astrocytoma matching to methylation class oligosarcoma following embolization, a case report
Source: Acta Neuropathol Commun. 2024 Dec 20;12:196. doi: 10.1186/s40478-024-01908-7 (PMC11662476; doi:10.1186/s40478-024-01908-7)

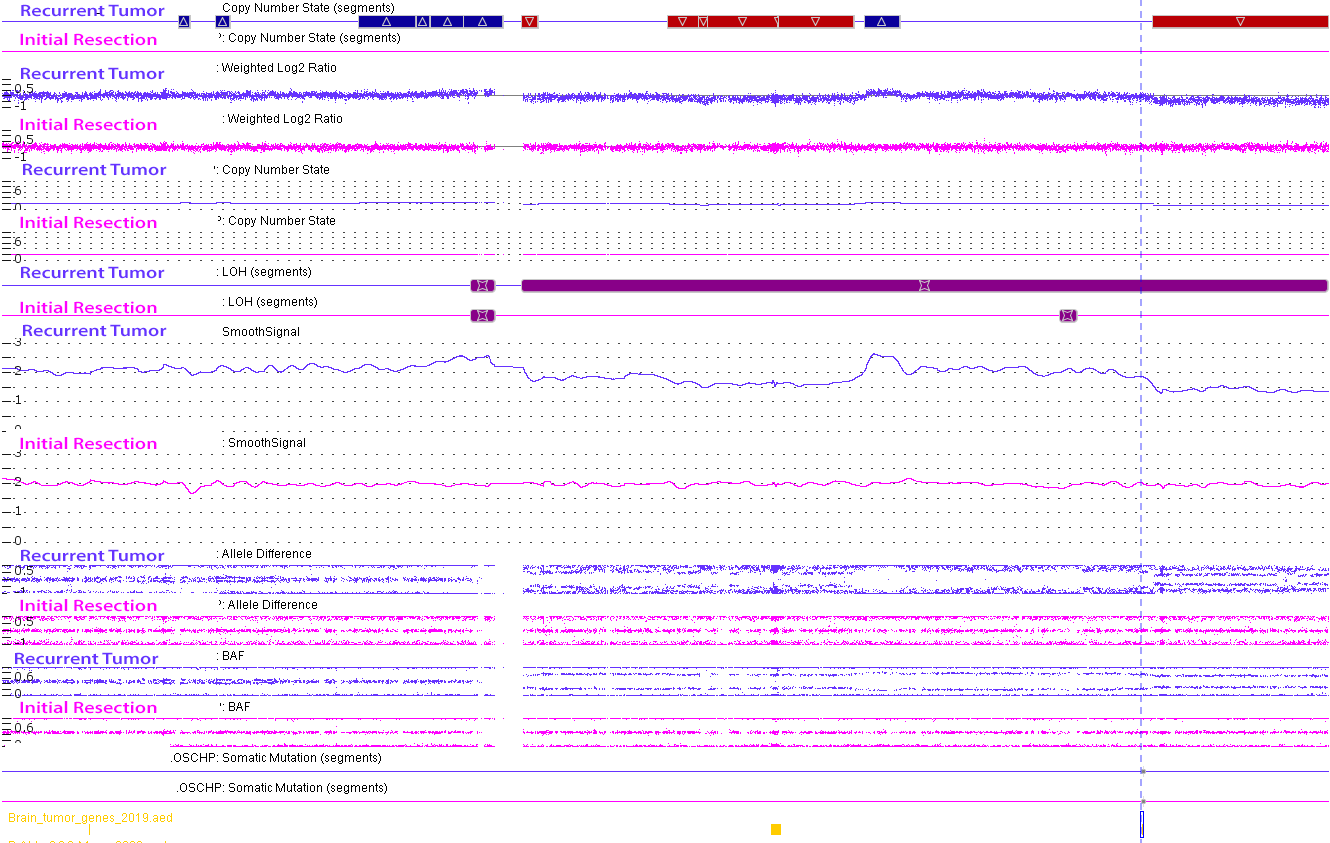

Supplement: Supplementary file 1 — Additional file 1. [file 40478_2024_1908_MOESM1_ESM.png]
